# Comparison of Infectious Complications with BCMA-directed Therapies in Multiple Myeloma

**DOI:** 10.21203/rs.3.rs-3911922/v1

**Published:** 2024-02-07

**Authors:** Alexander Lesokhin, Karthik Nath, Tala Shekarkhand, David Nemirovsky, Andriy Derkach, Bruno Almeida Costa, Noriko Nishimura, Tasmin Farzana, Colin Rueda, David Chung, Heather Landau, Oscar Lahoud, Michael Scordo, Gunjan Shah, Hani Hassoun, Kylee Maclachlan, Neha Korde, Urvi Shah, Carlyn Rose Tan, Malin Hultcrantz, Sergio Giralt, Saad Usmani, Zainab Shahid, Sham Mailankody

**Affiliations:** Memorial Sloan Kettering Cancer Center; Memorial Sloan Kettering Cancer Center; Memorial Sloan Kettering Cancer Center; Memorial Sloan Kettering Cancer Center; MSKCC; Memorial Sloan Kettering Cancer Center; Memorial Sloan Kettering Cancer Center; Memorial Sloan Kettering Cancer Center; Memorial Sloan Kettering Cancer Center; Memorial Sloan Kettering Cancer Center; Memorial Sloan-Kettering Cancer Center; Memorial Sloan Kettering Cancer Center; Memorial Sloan Kettering Cancer Center; MSKCC; Memorial Sloan Kettering Cancer Center; Memorial Sloan Kettering Cancer Center; korden@mskcc.org; Memorial Sloan Kettering Cancer Center; Memorial Sloan Kettering Cancer Center; Memorial Sloan Kettering Cancer Center; Memorial Sloan-Kettering Cancer Center.; Memorial Sloan Kettering Cancer Center; Memorial Sloan Kettering Cancer Center; Memorial Sloan Kettering Cancer Center

## Abstract

B-cell-maturation-antigen (BCMA)-directed therapies are highly active for multiple myeloma, but infections are emerging as a major challenge. In this retrospective, single-center analysis we evaluated infectious complications after BCMA-targeted chimeric-antigen-receptor T-cell therapy (CAR-T), bispecific-antibodies (BsAb) and antibody-drug-conjugates (ADC). The primary endpoint was severe (grade ≥ 3) infection incidence. Amongst 256 patients, 92 received CAR-T, 55 BsAb and 109 ADC. The incidence of severe infections was higher with BsAb (40%) than CAR-T (26%) or ADC (8%), including grade 5 infections (7% vs 0% vs 0%, respectively). Comparing T-cell redirecting therapies, the incidence rate of severe infections was significantly lower with CAR-T compared to BsAb at 1-year (incidence-rate-ratio [IRR] = 0.43, 95%CI 0.25–0.76, P = 0.004). During periods of treatment-emergent hypogammaglobulinemia, BsAb recipients had higher infection rates (IRR:2.27, 1.31–3.98, P = 0.004) and time to severe infection (HR 2.04, 1.05–3.96, P = 0.036) than their CAR-T counterparts. During periods of non-neutropenia, CAR-T recipients had a lower risk (HR 0.44, 95%CI 0.21–0.93, P = 0.032) and incidence rate (IRR:0.32, 95% 0.17–0.59, P < 0.001) of severe infections than BsAb. In conclusion, we observed an overall higher and more persistent risk of severe infections with BsAb. Our results also suggest a higher infection risk during periods of hypogammaglobulinemia with BsAb, and with neutropenia in CAR-T recipients.

## INTRODUCTION

Treatments for relapsed/refractory multiple myeloma have greatly evolved over the last several years. This is especially the case for treatment modalities that target B-cell maturation antigen (BCMA), which is expressed on the surface of normal and malignant plasma cells (1). Therapeutic agents that target BCMA include antibody drug conjugates (ADC), and the T-cell redirecting therapies of chimeric antigen receptor T-cell therapy (CAR-T) and bispecific antibodies (BsAb) (2). There are now five US FDA approved CAR-T and BsAb therapies for relapsed/refractory multiple myeloma and many others in development. Four of these five agents target BCMA (3–7).

Despite their unprecedented response rates, there are emerging reports regarding the spectrum of infections associated with BCMA-directed T-cell redirecting therapies (8). With increasing use of T-cell redirecting therapies, it is important to better understand the risk and nature of infectious complications with these therapies, particularly in a patient population that is already highly susceptible to infections (9, 10). This in turn will help optimize infection treatment and mitigation strategies, and potentially aid with patient treatment selection as well.

In this study, we performed a retrospective analysis to assess the nature, incidence, rate, and risk factors for infectious complications in recipients of BCMA-directed CAR-T compared to BCMA-targeted BsAb in relapsed/refractory multiple myeloma. Infectious events with T-cell redirecting therapies were also compared to a similarly heavily pretreated population who received BCMA-directed ADCs.

## SUBJECTS and METHODS

This was a single-center, retrospective study conducted at Memorial Sloan Kettering Cancer Center (MSK) to compare infectious complications in patients receiving BCMA-targeting therapies. Adult patients ≥ 18-years of age with a diagnosis of relapsed/refractory multiple myeloma and treated with a BCMA-targeting CAR-T, BsAb or ADC were included in this analysis. This study was approved by the MSK institutional review board.

### Study population

BCMA-targeting therapies included a commercial or investigational autologous CAR-T, commercial or investigational BsAb, and commercial or investigational ADC. The date of CAR-T infusion (day 0) was between 03/22/2017–02/27/2023 for patients treated with BCMA-targeting CAR-T. The date of treatment initiation (day 0) was between 01/21/2020–02/07/2023 for patients treated with BCMA-targeting BsAb, and between 9/17/2018–01/07/2023 for patients treated with BCMA-targeting ADCs.

Within those that received T-cell redirecting therapies, patients may have received both a BsAb and CAR-T. However, in patients that received more than 1 BsAb, only the first BsAb was included, and infectious events after the 2nd BsAb were not included in this analysis. Similarly, in patients that received more than 1 CAR-T, infectious events only following the 1st CAR-T were included. Patients who received allogeneic or G protein–coupled receptor, class C, group 5, member D (GPRC5D)-targeting CAR-T, and those that received subtherapeutic doses of BsAb on dose escalation cohorts of clinical trials were excluded.

### Study endpoints

The primary endpoint was the incidence of severe (grade ≥ 3) infections. Secondary endpoints were evaluated in those that received T-cell redirecting therapies and included infection rate over time, infectious organisms, infectious sites, infection risk factors, and the impact of modifiable risk factors of treatment-emergent hypogammaglobulinemia and neutropenia on infectious events. All infection-specific events were collected from day 0 until the date of next line of therapy or last follow-up, with a data cut-off of 06/01/23. Adverse events were graded according to the Common Terminology Criteria for Adverse Events, version 5.0.

Cytokine release syndrome in the absence of a concurrent infectious process, even if treated with empiric antibiotics, was not considered an infectious event. Prophylactic antimicrobials were administered according to institutional and protocol guidelines. Hypogammaglobulinemia was defined as an IgG level < 400 mg/dL. In patients with IgG isotype multiple myeloma, functional hypogammaglobulinemia was calculated by subtracting out the monoclonal IgG paraprotein from the serum IgG. Neutropenia was defined as neutrophil count < 1000/mcL. Lymphopenia was defined as a lymphocyte count < 500/mcL. Granulocyte colony-stimulating factor (G-CSF) and intravenous immunoglobulin (IVIg) was administered at the discretion of the treating physician. Baseline laboratory values were obtained just prior to lymphodepleting chemotherapy in CAR-T recipients and just prior to treatment initiation in BsAb and ADC recipients.

### Statistical Analysis

The Wilcoxon rank sum test was used to assess differences in continuous demographic variables between treatment groups. Fisher’s exact test and Pearson’s Chi-squared test were used to assess differences in categorical variables. Rates of first infection were estimated using cumulative incidence curves with initiation of new treatment or death as a competing event. Cause-specific analysis using Cox proportional-hazard model was used to assess the association between infection risk and baseline pretherapy risk factors, as well as determining the association between infection risk and multiple myeloma treatment while adjusting for periods of neutropenia and periods of hypogammaglobulinemia post-treatment as time-dependent covariates (TDC). Poisson regression with the number of days on the follow-up window as offset was used to assess the association between rates of infection and the covariates during the first 100-days, first 6-months, and first year from start of the treatment. Poisson regression with a patient-specific random effect was used to assess the association between rates of infection and periods of neutropenia and periods of hypogammaglobulinemia post-treatment. The pretherapy covariates used in the multivariable Cox regression and multivariable Poisson models were age, triple/penta-refractory status, prior receipt of BCMA-targeted therapy, neutropenia, lymphopenia, and hypogammaglobulinemia. All statistical analyses were performed using R 4.2.2.

## RESULTS

### Patients

A total of 256 patients were included in this analysis, with 92 CAR-T, 55 BsAb and 109 ADC-treated patients. The baseline characteristics of patients that received T-cell redirecting therapies (CAR-T and BsAb) are summarized in Table 1. The median age of patients treated with CAR-T was 62 years (interquartile range [IQR]: 56–69 years) compared to 65 years (IQR: 58–72 years) in the BsAb treated group (P = 0.043). Patients treated with CAR-T received a median of 7 prior lines of therapy (IQR: 5–8) compared to a median of 6 prior lines of therapy (IQR: 4–9) with BsAb. Nearly all patients in the CAR-T arm had a prior autologous transplant (97%), compared to 75% in the BsAb arm (P < 0.001). Patients in the study were representative of the real-world population with 33% in the BsAb treated group having had prior exposure to CAR-T, whilst only 1% in the CAR-T cohort had prior BsAb exposure.

### Incidence and grade of severe infections in recipients of CAR-T and BsAb

The median follow-up duration for infection-specific events was similar in patients that received T-cell redirecting therapies, with a median follow-up of 5.8 months (IQR: 3.7–9.2 months) in the CAR-T arm compared to 4.3 months (IQR: 3.2–9.8 months) in the BsAb arm.

**Figure 1:**
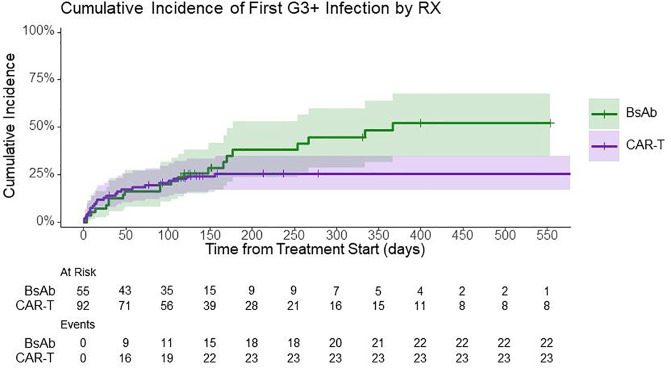
Estimated cumulative incidence of grade ≥3 infections over time.

A total of 214 infectious events were reported, with 115 after CAR-T and 99 after BsAb. Forty percent of CAR-T recipients and 27% of BsAb recipients had no infections reported during the follow-up period. Regarding the primary endpoint, the incidence of severe (grade ≥ 3) infections was numerically lower with CAR-T with 26% of patients experiencing severe infections compared to 40% with BsAb (hazard ratio [HR] 0.58, 95% confidence interval [CI] 0.32–1.04, P = 0.067) – Fig. 1.

After adjusting for pretherapy variables, the multivariable analysis demonstrated a lower risk for time to first severe infections with CAR-T compared to BsAb, but this did not reach statistical significance (HR 0.60, 95% CI 0.31–1.17, P = 0.14) – Table 2. Pretherapy variables including patient age, triple/penta-refractory status, prior receipt of BCMA-targeted therapy and baseline lymphopenia or hypogammaglobulinemia did not predict for severe infection on multivariable analysis in the entire cohort. However, within the CAR-T cohort, baseline lymphopenia (prior to lymphodepleting chemotherapy) was associated with a higher risk for severe infection (HR 2.82, 95% CI 1.18–6.72, P = 0.02). We did not assess for baseline neutropenia as there were very few patients with pretherapy neutropenia in both cohorts (baseline neutropenia in CAR-T: 1% and BsAb: 6%).

Of the 26% of CAR-T patients with severe infections, all experienced only grade 3 infections, and there were no grade 4 or 5 events in the CAR-T cohort. In the BsAb group, 40% experienced grade 3 infections, 4% had grade 4 infections and 7% experienced grade 5 infections – Table 3. Seven percent of patients in the CAR-T arm and 20% in the BsAb arm experienced > 1 severe infection.

In case dual exposure to both BsAb and CAR-T may have impacted the study findings, the 18 patients within the BsAb arm that received prior CAR-T were excluded, and the analysis was repeated for the primary endpoint. The baseline characteristics of this subset of patients is included in **Supplemental Table 1**. Of the 37 patients treated with BsAb and who did not receive prior CAR-T, the median follow-up duration was 4.7 months (IQR 3.5–10.9 months) during which there were a total of 81 infections reported. The incidence of ≥ 1 severe infection remained high at 46%, including 5% of patients experiencing grade 4 infections, and 8% having grade 5 infections.

Regarding any-grade infections in the entire cohort, there was no significant difference in the infection incidence between BsAb and CAR-T on both univariable and multivariable analysis – **Supplemental Table 2**. The median time to the first infectious event of any-grade was shorter at 2.5 months (95% CI 1.2 – not reached [NR]) post-CAR-infusion compared to 3.1 months (95% CI 1.2–5.5 months) after the initiation of BsAb.

### Comparison to severe infections with ADC

A cohort of 109 patients treated with BCMA-directed ADCs were used as a control to assess the infection risk with BCMA-directed ADCs in a similarly heavily pretreated patient population. The baseline characteristics of this patient population are provided in **Supplemental Table 3** and were overall comparable to both the CAR-T and BsAb groups with a median age of 67 years (IQR 61–73 years), median of 6 (IQR 5–8) prior lines of therapy and 78% having received a prior autologous stem cell transplant.

The median follow-up duration for infection-specific events was 3.8 months (IQR 1.4–8.7 months) during which time there was a total of 31 infections reported. The incidence of severe infections in patients treated with ADC was low at 8% - Table 3. There was 1 grade 4 infection, and 0 grade 5 infections. Pretherapy patient or disease characteristics did not predict for infection in this cohort.

### Infection rates over time in the CAR-T and BsAb recipients

In univariable analysis, there was no significant difference in the incidence rate of severe infections up to 100-days post CAR-T compared to 100-days post BsAb (incidence rate ratio [IRR]: 1.11, 95% CI 0.55–2.39, P = 0.8), and this was maintained in a multivariable analysis adjusting for the baseline covariates (IRR 1.58, 95% 0.68–3.89, P = 0.3). Similarly, there was no significant difference in incidence rate of severe infections between the two groups up to 6-months post treatment initiation. However, when extending the follow-up to 1-year, there was a significantly lower incidence rate for severe infections with CAR-T compared to BsAb (IRR 0.45, 95% CI 0.27–0.74, P = 0.002), and this was maintained in a multivariable analysis (IRR 0.43, 95% CI 0.25–0.76, P = 0.004) – Table 4. Notably, 79% of first severe infections occurred within day-100 post CAR-infusion in the CAR-T arm compared to only 50% occurring within the first 100 days after commencement of BsAb. Together, these findings suggest that the risk of severe infections is prolonged with BsAb compared to CAR-T.

Similar findings were also observed when assessing the incidence rate of any-grade infections between the two groups.

### Impact of treatment-emergent hypogammaglobulinemia on infections

**Figure 2:**
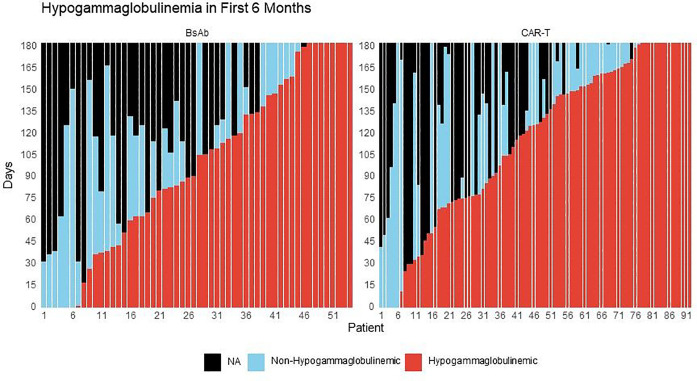
Functional serum IgG levels over time after treatment initiation up to 6 months of follow up. The x-axis represents individual patients. The colored bars report the number of days within the 1^st^ 6-months during which each patient experienced functional hypogammaglobulinemia (IgG < 400mg/dL) or non-hypogammaglobulinemia (IgG ≥ 400mg/dL).

The overall proportion of time that patients experienced treatment-emergent hypogammaglobulinemia during the follow-up period was similar between BsAb and CAR-T – Fig. 2. Despite the similar time periods of post-therapy hypogammaglobulinemia, CAR-T recipients appeared to have a lower incidence rate of severe infections compared to BsAb during periods of hypogammaglobulinemia, but this was not statistically significant at 6-months follow-up (IRR 0.61, 95% CI 0.33–1.14, P = 0.11) – **Supplemental Table 4A**. When extending the follow-up to 1-year, the difference was statistically significant (IRR 0.44, 95% CI 0.25–0.76, P = 0.004). Similar associations were found for any-grade infections when comparing CAR-T to BsAb during periods of hypogammaglobulinemia (IRR at 6 months 0.65, 95% CI 0.45–0.95, P = 0.025: IRR at 12 months 0.55, 95% CI 0.40–0.77, P < 0.001). During periods of non-hypogammaglobulinemia, there also appeared to be a lower rate of severe infections at 1-year with CAR-T compared to BsAb, but this was not statistically significant (IRR 0.46, 95% CI 0.13–1.54, P = 0.2) – **Supplemental Table 4B.**

The impact of treatment-emergent hypogammaglobulinemia was also assessed as a risk factor for time- to-infection. Within both cohorts, the presence of hypogammaglobulinemia appeared to associate with incidence of severe infections, but this was not statistically significant (CAR-T - HR 1.44, 95% CI 0.53–3.93, P = 0.5; BsAb - HR 1.37, 95% CI 0.46–4.07, P = 0.6) – **Supplemental Table 5A**. However, when focusing only within periods of post-therapy hypogammaglobulinemia, the incidence of severe infections in the CAR-T cohort was significantly lower compared to BsAb (HR 0.49, 95% CI 0.25–0.96, P = 0.036). There was no significant difference in severe infection risk during periods of non-hypogammaglobulinemia (HR 0.77, 95% CI 0.21–2.78, P = 0.7) – **Supplemental Table 5B**. Similarly, during periods of post-therapy hypogammaglobulinemia, patients in the CAR-T cohort had significantly lower incidence of any-grade infections compared to BsAb (HR 0.51, 95% CI 0.31–0.83, P = 0.007) – **Supplemental Table 5C**. Together, these results suggest that periods of hypogammaglobulinemia may have a more profound impact on severe infection risk with BsAb as compared to CAR-T. Notably, of the 7% of patients within the BsAb arm that experienced grade 5 infections, the most recent IgG level available prior to the onset of the grade 5 infectious event was < 200mg/dL in all patients.

### Impact of treatment-emergent neutropenia on infections

**Figure 3:**
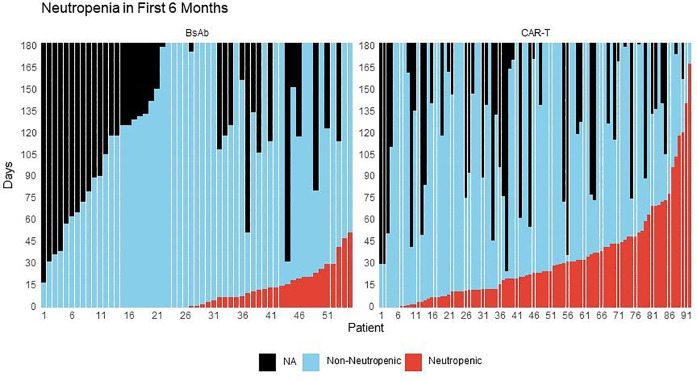
Absolute neutrophil count over time after treatment initiation up to 6 months of follow up. The x-axis represents individual patients. The colored bars report the number of days within the 1^st^ 6-months during which each patient experienced neutropenia (neutrophil count < 1000/mcL) or non-neutropenia (neutrophil count ≥ 1000/mcL).

Post-therapy neutropenia was more common in recipients of CAR-T compared to BsAb recipients – Fig. 3. The presence of neutropenia was associated with a significantly higher incidence rate of severe infections with CAR-T when compared to periods of non-neutropenia (IRR 2.68, 95% CI 1.14–6.31, P = 0.024) in the first 100-days post treatment initiation. There was also a higher incidence rate of severe infections during periods of neutropenia with BsAb within the first 100 days, but this was not statistically significant (IRR 3.45, 95% CI 0.75–16.00, P = 0.11). Given as severe infections predominantly occurred in the first 100-days post treatment initiation with CAR T-cell therapy, this suggests that periods of neutropenia during the first 100-days may have a more profound impact on severe infection rate in the setting of CAR-T. Periods of post-therapy neutropenia continued to be associated with a significantly higher incidence rate of severe infection at follow-up that extended to 6-months (IRR 3.40, 95% CI 1.47–7.83, P = 0.004) and 1-year in CAR-T recipients (IRR 4.32, 95% CI 1.85–10.1, P < 0.001). For recipients of BsAb, the presence of neutropenia at 6-months (IRR 2.93, 95% CI 0.93–2.93, P = 0.066) and 1-year (IRR 2.85, 95% CI 1.02–7.96, P = 0.046) also appeared to affect the rate of severe infections and reached significance at 1-year follow-up – **Supplemental Table 6A**. We also tested for differences in the incidence rate for severe infections within periods of post-therapy neutropenia in the CAR-T compared to BsAb cohort and found that the incidence rate was lower with CAR-T, but this difference was not statistically significant (IRR at 100-days follow-up: 0.67, 95% CI 0.18–4.30, P = 0.6) – **Supplemental Table 6B**.

Notably, during periods of post-therapy non-neutropenia at 1-year follow-up, CAR-T recipients had a significantly lower infection rate compared to BsAb (IRR 0.32, 95% CI 0.17–0.59, P < 0.001) – **Supplemental Table 6C**. Similarly, during periods of post-therapy non-neutropenia, CAR-T recipients had a significantly lower time to infection compared to BsAb recipients (HR 0.44, 95% CI 0.21–0.93, P = 0.032) – **Supplemental Table 7A.**

### Infection types

In CAR-T recipients, the proportion of any-grade bacterial, viral, fungal, and parasitic infections was 49%, 48%, 4% and 1%, respectively. In BsAb recipients, the proportion of bacterial, viral, fungal, and parasitic infections was 53%, 42%, 3% and 0, respectively. The two grade 4 infections in the BsAb arm were COVID-19 infection and Stenotrophomonas maltophilia pneumonia. Of the four grade 5 infections in the BsAb cohort, three were bacterial pneumonia (1 Klebsiella pneumoniae, 2 Pseudomonas aeruginosa) and one bacteremia (Klebsiella aerogenes). Grade 5 events occurred between day 30 to day 334 of BsAb initiation.

Infections were most common in the respiratory tract in both cohorts. The proportion of bloodstream, upper respiratory, lower respiratory, gastrointestinal, genitourinary, skin/soft tissue, and other infections was 15%, 40%, 14%, 16%, 11%, 7% and 3%, respectively in the CAR-T arm compared to 9%, 49%, 13%, 9%, 9%, 4% and 5%, respectively in the BsAb treated arm. Grade ≥ 3 opportunistic infections or viral reactivation syndromes accounted for 1% (1 cytomegalovirus reactivation) of all CAR-T infections, and 3% of BsAb infections (1 Pneumocystis pneumonia, 1 cytomegalovirus reactivation and 1 herpes simplex viral infection). Further details of the infection site, grades and organism are provided in **Supplemental Tables 8A and 8B**.

## DISCUSSION

Whilst there have been early reports regarding infection risks with BCMA-directed T-cell redirecting therapies, there is currently a paucity of published data comparing infectious complications in larger cohorts of patients treated with distinct T-cell redirecting strategies (11). In this retrospective analysis of 147 patients that received T-cell redirecting therapies, we observed that the incidence of severe (grade ≥ 3) infections was higher in similar patient populations treated with BCMA-targeted BsAb compared to BCMA-targeted CAR-T. No patients in the CAR-T cohort experienced grade 4–5 infections, whereas grade 4 and 5 events were observed in 4% and 7% of BsAb recipients, respectively. Furthermore, similar to prior smaller studies, we found that the frequency and pattern of infections with BsAb and CAR-T was much higher than in a similarly heavily pretreated population of 109 patients who received BCMA-targeting ADC (12).

Within those that received T-cell redirecting therapies, there was a significantly higher incidence rate for severe infections with BsAb compared to CAR-T at extended follow-up to 1-year. Pretherapy risk factor of lymphopenia associated with a higher risk for severe infection in CAR-T recipients, and pretherapy hypogammaglobulinemia did not predict for subsequent infections. We also assessed for the impact of modifiable risk factors of post-therapy hypogammaglobulinemia and neutropenia on infection risk. During periods of treatment-emergent hypogammaglobulinemia, CAR-T recipients appeared to have both a lower incidence, and incidence rate of severe infections when compared to recipients of BsAb. These results suggest that BsAb recipients have a higher infection risk than their CAR-T counterparts, and that during periods of hypogammaglobulinemia the incidence rate for severe infections is more pronounced after BsAb compared to CAR-T. Treatment-emergent neutropenia was more pronounced after CAR-T, and CAR-T recipients had a lower infection risk in compared to BsAb recipients during periods of non-neutropenia.

Despite CAR-T, BsAb and ADC having the same BCMA target, our analysis highlights the potential differences between differing classes of T-cell redirecting therapies regarding the risk for severe infections. Patients receiving BCMA-BsAb should be considered a higher-risk group compared to their CAR-T counterparts. Although the risk for severe infections appeared to decline over time in CAR-T recipients, the infection risk is more persistent with BsAb. Whether or not this relates to chronic stimulation of T-cells and their subsequent exhaustion from continuously dosed BsAb agents, or an earlier immune reconstitution with CAR-T remains unclear (13, 14). Furthermore, the infection risk was much lower after BCMA-ADCs than both T-cell redirecting therapies, and again suggesting that infection risks could be more attributable to the mechanism of action of specific classes of therapies rather than inherent patient characteristics or the target protein only. Whether or not infection risk may correlate with the potency of such therapies is best answered in a randomized setting.

There were 49% bacterial, 48% viral, and 4% fungal infections in the CAR-T cohort, and infections were predominantly of the upper or lower respiratory tract (54%). The observed proportion of infection types and locations were consistent with other previously published reports from recipients of BCMA CAR-T (15). Similarly, as has been reported in prior smaller studies, there was a predominance of bacterial (53%) and viral infections (42%) after BCMA BsAb (11, 16). We reported an overall low number of opportunistic infections and viral reactivation syndromes in both treatment arms.

Though speculative, the presence of baseline lymphopenia predicting for severe infection in CAR-T recipients may relate to the differential impact of bridging therapies on immune function. Another important question that we addressed was the impact of treatment-emergent hypogammaglobulinemia on infection risk. Hypogammaglobulinemia is an expected on-target-off-tumor toxicity of BCMA-directed therapies given BCMA expression on normal and malignant plasma cells (17). After correcting for monoclonal immunoglobulin production in patients with IgG myeloma, we evaluated the impact of treatment-emergent moderate-severe hypogammaglobulinemia (IgG < 400 mg/dL) on infection risk. Overall, the proportion of hypogammaglobulinemia appeared similar between CAR-T and BsAb recipients. However, during periods of post-therapy hypogammaglobulinemia, there appeared to be a higher risk of severe infections with BsAb compared to CAR-T. Consistent with prior reports and recent consensus guidelines, these data suggest that immunoglobulin replacement should be preferentially considered in the setting of treatment-induced hypogammaglobulinemia for recipients of BsAb (18–20).

Periods of treatment-induced neutropenia were more common after CAR-T compared to BsAb. This is consistent with the high rates of hematologic toxicity that have been reported following autologous CAR-T, including some cases of prolonged and severe cytopenias (21–23). During periods of non-neutropenia, there was a lower infection rate and time to infection in recipients of CAR-T compared to BsAb, supporting the consideration of G-CSF use in this setting.

Our study has several limitations. When compared to prior retrospective analyses, our study is relatively large with 147 patients treated CAR-T and BsAb, and a control group of 109 patients treated with ADC. Nonetheless, this is a retrospective analysis of a heterogenous patient population that included both investigational and commercial therapies. The retrospective nature may not have captured all infections, particularly if non-emergent, and respiratory viral polymerase chain reaction testing may not have been uniformly performed leading to possible under-reporting (24). Potential variations in antimicrobial prophylaxis also makes it less straightforward to generalize our results. Although there was likely consistency in antiviral and pneumocystis jiroveci pneumonia prophylaxis where routine prophylaxis is typically recommended in both CAR-T and BsAb recipients, it is possible that recommendations for antibacterial prophylaxis may have changed over the study period. The follow-up duration for infection-specific events was not extensive, and ideally, our study findings would require prospective validation in an even larger cohort, particularly regarding the finding of differential effects of post-therapy hypogammaglobulinemia and non-neutropenia on infection risk between the two cohorts. We excluded patients that received suboptimal doses of BsAb on dose-escalation trials, but there may be variation in terms of dosing schedules which could have impacted infection risk. Although we focused our analysis on correctable factors of treatment-emergent neutropenia and hypogammaglobulinemia, the complex effects of T-cell redirecting therapies on immunity and infection risk may be better predicted with other immune monitoring tools, including assessment of post-treatment lymphocyte subsets (25). We also did not analyze the effect of immunoglobulin replacement in this study. This is because of the variation in IVIg use over the study duration, and variations in the frequency of administration in those that received it. Instead, we chose to focus on treatment-induced functional immunoglobulin deficiency regardless of IVIg use, as there would be correction of hypogammaglobulinemia during time periods of appropriate IVIg supplementation. Moving forward, it is crucial to prospectively evaluate IVIg administration on infection risk in BCMA-directed BsAb recipients to assess whether, and to what degree, IVIg supplementation could mitigate the risk of severe infections. Although beyond the scope of our study, as more products are becoming US FDA approved, it would also be important to comprehensively ascertain and compare infection risks between T-cell redirecting therapies that target other proteins in multiple myeloma (16, 26–28).

In conclusion, we report an increased susceptibility to severe infections, and more persistent infection risk with BCMA-directed BsAb compared to CAR-T. We also observed a much lower risk for severe infections in a similar patient population treated with BCMA-directed ADCs. Within those that received T-cell redirecting therapies, our results suggest a higher infection risk with BsAb during periods of hypogammaglobulinemia and periods of non-neutropenia than their CAR-T counterparts. Together, these findings suggest that distinct supportive care strategies are clinically relevant for these treatment modalities.

## Figures and Tables

**Table 1:** Baseline characteristics of patients who received T-cell redirecting therapies.

Characteristic	Cohort		p-value^2^
	BsAb, N = 55^1^	CAR-T, N = 92^1^	
Age	65 (58, 72)	62 (56, 69)	**0.043**
Isotype			> 0.9
Kappa	31 (56)	52 (57)	
Lambda	24 (44)	40 (43)	
Refractory status			**0.046**
Neither	6 (11%)	20 (22%)	
Triple	49 (89)	72 (78)	
Penta-	30 (55)	32 (35)	
No. of prior lines	6.0 (4.0, 9.0)	6.5 (5.0, 8.2)	0.7
Prior BCMA	25 (45%)	12 (13%)	**< 0.001**
Prior ADC	19 (35%)	11 (12%)	**0.001**
Prior CAR-T	18 (33%)	0 (0%)	**< 0.001**
Prior Auto-SCT	41 (75%)	89 (97%)	**< 0.001**
Prior Allo-SCT	3 (5.5%)	5 (5.4%)	> 0.9
Neutropenia ≤ 1000/mm^3^	3 (5.5%)	1 (1.1%)	0.15
Lymphopenia ≤ 500/mm^3^	21 (38)	21 (23)	0.046
Hypogammaglobulinemia	26 (49%)	55 (60%)	0.2

1 Median (IQR); n (%)

2 Wilcoxon rank sum test; Pearson’s Chi-squared test; Fisher’s exact test

BsAb, bispecific antibodies; BCMA, B-cell maturation antigen; CAR-T, chimeric antigen receptor T-cell therapy; PR, partial response; SD stable disease; PD, progressive disease; ADC, antibody drug conjugate; Auto-SCT, autologous stem cell transplant; Allo-SCT, allogeneic stem cell transplant.

**Table 2:** Multivariable cause-specific Cox regression of baseline characteristics for time to first severe infections

Characteristic	N	Event	HR	95% CI	P-value

Cohort	53	21	-	-	0.14
BsAb	92	24	0.60	0.31, 1.17	
CAR-T					

Age	145	45	0.99	0.96, 1.02	0.5

Refractory Status	25	7	-	-	0.8
Neither	59	18	0.91	0.36, 2.32	0.6
Triple	61	20	1.29	0.50, 3.29	
Penta-					

Prior BCMA therapy	145	45	0.65	0.28, 1.48	0.3

Neutropenia ≤ 500/mm	145	45	1.78	0.37, 8.46	0.5

Lymphopenia ≤ 500/mm	145	45	1.80	0.92, 3.53	0.086

Hypogammaglobulinemia	145	45	1.11	0.59, 2.07	0.8

BsAb, bispecific antibodies; CAR-T, chimeric antigen receptor T-cell therapy; BCMA, B-cell maturation antigen; HR, hazard ratio; CI, confidence interval.

**Table 3:** Primary study endpoint of severe (grade ≥ 3) infections

	BsAb, N = 55	CAR-T, N = 92	ADC, N = 109

Infection grade	22 (40)	24 (26)	9 (8)
- grade ≥ 3 infections	2 (4)	0	1 (1%)
- grade 4 infections	4 (7)	0	0
- grade 5 infections			

Recurrent severe infections	11 (20)	6 (7)	2 (2%)

n (%)			

BsAb, bispecific antibodies; CAR-T, chimeric antigen receptor T-cell therapy; ADC, antibody drug conjugate.

**Table 4:** Multivariable Poisson regression of severe infections up to the 1st year follow-up post-therapy

Characteristic	IRR	95% CI	p-value
Cohort			
BsAb	–	–	
CAR-T	0.43	0.25, 0.76	**0.004**
Age	0.99	0.96, 1.01	0.3
Triple/Penta Refractory			
Neither	–	–	
Triple	0.90	0.41, 2.18	0.8
Penta	1.40	0.66, 3.33	0.4
Prior BCMA-directed therapy	0.62	0.29, 1.26	0.2
Neutropenia ≤ 500/mm^3^	0.92	0.22, 2.62	0.9
Lymphopenia ≤ 500/mm^3^	1.63	0.92, 2.85	0.089
Hypogammaglobulinemia	1.24	0.73, 2.12	0.4

IRR, incidence rate ratio; CI, confidence interval; BsAb, bispecific antibodies; CAR-T, chimeric antigen receptor T-cell therapy; BCMA, B-cell maturation antigen.

## Data Availability

Subject to patient privacy and confidentiality obligations, access to patient-level data and supporting clinical documents will be available upon request to the corresponding author by email (lesokhia@mskcc.org). Any data and materials that can be shared will be released via a material transfer agreement and/or data access agreement.
